# Caregiving and Caregiver Health 1 Year into the COVID-19 Pandemic (CUIDAR-SE Study): A Gender Analysis

**DOI:** 10.3390/ijerph19031653

**Published:** 2022-01-31

**Authors:** María Del Río-Lozano, Mar García-Calvente, Belén Elizalde-Sagardia, Gracia Maroto-Navarro

**Affiliations:** 1 Escuela Andaluza de Salud Pública (EASP), 18080 Granada, Spain; maria.rio.easp@juntadeandalucia.es (M.D.R.-L.); gracia.maroto.easp@juntadeandalucia.es (G.M.-N.); 2 Instituto de Investigación Biosanitaria de Granada ibs.Granada, 18012 Granada, Spain; 3 Departamento de Salud del Gobierno Vasco, Delegación de Salud de Gipuzkoa, 20010 San Sebastián, Spain; gbelizalde2@gmail.com

**Keywords:** COVID-19, informal care, caregiver, gender, health, self-perceived health, Spain

## Abstract

The COVID-19 pandemic has highlighted the importance of informal care and shown that women continue to shoulder the brunt of responsibilities in this area. In this study, we analyzed differences in caregiving and self-perceived health in a group of informal male and female caregivers 1 year into the COVID-19 pandemic. We performed a cross-sectional survey of 261 informal caregivers (165 women and 96 men) in two regions of Spain using computer-assisted telephone interviewing between February and April 2021. We performed descriptive, bivariate, and multivariate analyses to calculate the odds of poor self-perceived health according to different caregiver, care recipient, and caregiving characteristics. We also analyzed the perceived effects of the pandemic on caregiving, caregiver health, and other aspects of life. Compared with male caregivers, female caregivers were more likely to experience increases in caregiving intensity and burden and a decline in self-perceived health as a result of the pandemic. Men providing high-intensity care, however, also reported deteriorated health. Men experienced fewer reductions in informal support, a factor that exerted a protective health effect. Women, by contrast, experienced a reduction in all support systems and in this case, a third-level education exerted a protective effect. Our results provide key insights that should be taken into account to design gender-based interventions aimed at supporting already stretched and burdened caregivers. A greater sharing of responsibilities and more resources are needed.

## 1. Introduction

The COVID-19 pandemic caused by the SARS-COV-2 coronavirus has brought about a revolution in almost all dimensions of the life of humanity as a whole. The quick spread of the virus has led to a considerable increase in the need for care, which has been repositioned as the core element that sustains our lives.

The pandemic has had an unprecedented impact on health and social care systems and has brought to the fore the crucial role played by caregivers. Reduced access to formal care and support services for dependent persons and their carers has led to an increase in the number of people being cared for at home, with women shouldering a disproportionate share of the responsibilities and being exposed to a greater risk of COVID-19 [1,2]. Numerous international organizations have reported an increase in gender differences in caregiving intensity as a result of the pandemic and have called for gender-responsive actions to avoid undoing progress already made towards equality [3,4,5,6].

A number of studies have analyzed the differential health effects of the COVID-19 pandemic on men and women [7], with evidence showing that the accumulation of caregiving responsibilities and household chores has taken a toll on the mental health of women [8,9,10].

Most studies of how the pandemic has affected caregiver health have focused on frontline health professionals and formal (paid) carers [11], with little attention to the effects on informal caregivers, many of whom were already overstretched or overburdened before the pandemic [12]. We understand by informal caregivers those who provide care to dependent people in their immediate social network, such as family, friends, and neighbors, and who do not receive financial remuneration for the help they offer [13].

Research into the effects of the pandemic on the health of informal caregivers has mainly involved comparisons between caregivers and non-caregivers and has shown that the former are more likely to experience intensification of social isolation, anxiety, depression, fatigue, sleep disorders, and financial difficulties [14,15,16]. Somatic problems such as headache, body aches, and abdominal discomfort have also been found to be more common in long-term caregivers compared with short-term caregivers and non-caregivers [15]. Studies of the impact of the pandemic on caregivers to people with dementia and other neurodegenerative diseases have been particularly numerous and have shown negative consequences for emotional wellbeing [17,18,19,20]. Few studies, however, have analyzed diverse groups of informal caregivers looking after people with very different needs, and even fewer have explored the effects through the lens of gender.

Restricted access to social care and support services and disruptions to personal support networks during early lockdown increased the intensity of caregiving responsibilities in many homes. The situation was further exacerbated by difficulties accessing medical care for both caregivers and recipients, resulting in an intensification of stress, burden, social isolation, and mental health difficulties [21]. Sex-disaggregated data, however, are largely missing from the above studies, making it difficult to explore the impact of the pandemic on the experiences and health of informal caregivers from a gender perspective.

As in other Mediterranean European countries, informal care is very common in Spain, with over 50% of caregivers dedicating more than 20 h a week to this activity [22]. Previous findings from the research team have shown that caregiving is more likely to have negative health, social, and work-related effects on women [23,24]. Support from formal and informal networks throughout the care process has also been shown to have a strong influence on health and wellbeing [25,26,27].

The need for studies exploring caregiver experiences, behaviors, and coping strategies over time and from a gender perspective has become increasingly evident in recent years [28]. Our study provides the necessary framework for analyzing the effects of the COVID-19 pandemic on the health and experiences of men and women providing informal care in two regions of Spain. The participants in this study are long-term caregivers providing high-intensity care, thereby constituting a group that is particularly vulnerable to the effects of the pandemic and in need of support.

The aim of this study was to analyze differences in care provision and self-perceived health among informal male and female caregivers 1 year into the COVID-19 pandemic. We specifically investigated the characteristics of care and the perceived consequences of the pandemic associated with the perceived health of caregivers in both sexes.

## 2. Materials and Methods

We conducted a cross-sectional epidemiological study in 2021 of informal adult caregivers in two geographic regions of Spain: Granada in Andalusia and Gipuzkoa in the Basque Country. This was the first wave of the CUIDAR-SE survey to be conducted since the start of the COVID-19 pandemic.

CUIDAR-SE is the acronym derived from the terms care and follow-up (*cuidar* and *seguimiento* in Spanish), the abbreviated name of the project entitled “Longitudinal study of consequences of informal care in women and men caregiver’s in Andalusia and the Basque Country”. This project was designed to analyze the effects of caregiving on different aspects of life, including health, in a sample of men and women providing informal care in Spain.

The study population comprised informal caregivers, defined as men and women aged at least 18 years living in a family home who provided unpaid care to a dependent person (co-resident or not) and were registered as carers with the corresponding authorities.

The CUIDAR-SE study was started in 2013 and the participants were selected by multistage randomized cluster sampling using municipalities as primary units, census sectors as secondary units, and caregivers as final units. The sampling units were stratified to reduce the effects of the study design. Caregivers were stratified by gender and municipalities by size; allocation was proportional.

After excluding members of the cohort who had exited since the last wave of the survey conducted in 2019, the sample comprised 261 caregivers: 165 women and 96 men. Reasons for exits included death (caregiver or recipient), admission to a nursing home or hospital (care recipient), and discontinuation of caregiving for another reason.

The survey was performed using computer-assisted telephone interviewing (CATI) between February and April 2021. Telephone, rather than home, interviews were chosen because of the pandemic. The ad hoc structured questionnaire used in previous waves of the survey [29] was adapted to the CATI system. A new section was added to explore different dimensions of the COVID-19 pandemic and their impact on self-perceived caregiver health and other aspects of life. The questionnaire was piloted among 60 members of the cohort to ensure the comprehensibility of each item and appropriate interview duration.

The dependent variable was self-perceived general health, which was categorized as good (including good/excellent) or poor (fair/poor/very poor). The independent variables used for descriptive, explanatory, or adjustment purposes were as follows:

- Sociodemographic caregiver characteristics: gender, place of residence (Granada or Gipuzkoa), age, level of education, paid employment (yes/no), living with a partner (yes/no), and perceived social support (low/high) assessed using the Duke Social Support Index with 11 items validated for use in the Spanish population.
- Social and health-related care recipient characteristics: age, relationship with caregiver (spouse/partner, child, parent, other), caregiver-reported level of dependence (moderate, severe, major), cognitive impairment (yes/no), COVID-19 diagnosis in past 12 months (yes/no).
- Caregiving characteristics: type of tasks (personal care, physical mobility, household chores): yes/no and if yes, with or without help; caregiving intensity: hours a day spent on care (<8, 8–14, >14); level of caregiver burden (measured using the 22-item Zarit Burden Interview with a total possible score of 22 to 110, categorized as no burden (≤46), mild burden (47–55), or high burden (≥56); informal support: substantial or very substantial help with caregiving or household tasks (paid help) (yes/no) and substantial or very substantial help from family members or close social circles (unpaid help) (yes/no); formal support: receipt of the family caregiving allowance (PECF: *prestación económica por cuidados en el entorno familiar* in Spanish is a compensation measure that can be requested within the framework of the Dependency Law [30], addressed to dependent people who are cared for by a relative, when certain conditions are met in the caregiving, coexistence, and habitability of the house) in the past 12 months (yes/no) and use of home help services or home medical or nursing services in the past 12 months (yes/no in both cases).
- Perceived effects directly attributable to the COVID-19 pandemic on caregiving and other personal circumstances (yes/no in all cases): increase in caregiving intensity, increase in level of caregiver burden, reduction in informal support, reduction in formal support (allowances and services), negative impact on emotional wellbeing, finances, family relationships, and social life and leisure activities. Caregivers were also asked if they had been diagnosed with COVID-19 in the past 12 months.

Those selected for inclusion were sent a letter explaining the purpose of the study and inviting them to participate. They were then contacted by telephone to confirm their participation and arrange an interview. Interviews were conducted by trained personnel after obtaining informed consent. The study was approved by the Research Ethics Committee of Granada and the Research Ethics Committee of Euskadi. The CUIDAR-SE study and its methodology have been described in detail elsewhere [25,29].

A gender-stratified descriptive analysis was performed for all variables using absolute and relative frequencies. The association between gender and each variable was explored using the chi-square test, with statistical significance set at *p ≤* 0.05.

Bivariate analysis was used to determine the prevalence of poor self-perceived health in relation to each of the independent variables. The respective associations were analyzed by logistic regression analysis with adjustment for age.

Multivariate logistic regression analysis with calculation of odds ratios (ORs) was performed to determine the likelihood of poor self-perceived health according to caregiver and care recipient characteristics, type of care provision, and perceptions of the impact of the pandemic on different aspects of caregiving and personal circumstances. A combined model including male and female caregivers with adjustment for all other variables was also built to analyze the association between gender and self-perceived health. Forward stepwise selection was used to add variables shown to be significant in the bivariate analysis and other relevant variables from the theoretical model (caregiver–recipient relationship, cognitive impairment, and performance of personal care tasks). Independent variables that correlated with each other or with poor self-perceived health were excluded, namely, decline in emotional wellbeing as a result of the pandemic, caregiving intensity, and caregiver burden). Variance inflation factor analysis ruled out multicollinearity between the variables included. The same variables were used to build separate models for male and female caregivers in order to explore factors associated with self-perceived health in men and women. The magnitude of association in the three models was estimated using ORs with a confidence interval (CI) of 95%. All analyses were performed in SPSS.

## 3. Results

The characteristics of male and female caregivers are summarized in Table 1. Men were older, mostly retired, and cared for their wives (60%). Women, by contrast were more likely to be middle-aged (45–64 years) and mostly cared for parents, followed by children and partners. Significant gender differences were observed for caregiver age, care recipient age, and relationship between caregiver and care recipient.

### 3.1. Gender Differences in Care Provision and Perceived Effects of the COVID-19 Pandemic

Caregiving characteristics are summarized in Table 2, which clearly shows the intensive nature of the care provided by both women and men: caregiving duties accounted for 8–14 h of the day for 53% of women and 46% of men and >14 h of the day for 27% of women and 19% of men. High caregiver burden scores were observed in 29% of women and 24% of men (nonsignificant difference). The vast majority of caregivers (89% of men and 90% of women) performed personal care tasks, but a significantly higher proportion of women did so without help (61% vs. 45% for men). A similar pattern was observed for care-related household chores. Physical mobility tasks were performed more often by men, both with and without help. Significant gender differences were observed for the three types of tasks.

No significant differences were observed between men and women in terms of support received. Most caregivers, regardless of gender, stated that they received support from their families or close social circles, and very few had paid help. The most widely used formal support mechanism was the PECF allowance (received by 55% of women and 45% of men). This was followed by home medical and nursing services (44%, 39%) and home help services (19%, 24%).

Caregiver perceptions of the effects of the COVID-19 pandemic on their caregiving situation and other aspects of their lives in the past 12 months are summarized in Table 3. A significantly higher proportion of women reported an increase in caregiving intensity (44% vs. 27%) and a reduction in informal support (37% vs. 21%). Women also felt that their level of burden had increased but in this case the difference with men was not significant. No differences were observed for changes to formal support services, with 80% of men and 82% of women reporting that they were the same as before the pandemic.

A significantly higher proportion of women stated that the pandemic had had a negative effect on their emotional wellbeing (75% vs. 56% for men). The pandemic had also affected the caregivers’ finances and family and social relationships, but the differences between men and women were not significant. More than half of those interviewed, regardless of gender, reported a worsening of social life and leisure activities. Finally, 8% of male caregivers and 7% of female caregivers had been diagnosed with COVID-19 in the previous 12 months.

### 3.2. Poor Self-Perceived Health According to Caregiving, Caregiver, and Care Recipient Characteristics

Overall, 45% of female caregivers and 30% of male caregivers described their health as fair, poor, or very poor (Table 1). The results of the bivariate analyses adjusted for age are shown in Table 4, which shows the prevalence of poor self-perceived health according to each of the study variables by gender. It also shows the respective associations. After adjustment for age, women were almost twice as likely as men to describe their health as poor, and the association was significant (OR: 1.96; CI: 1.28–3.39).

In the gender-stratified analysis, poor self-perceived health was not significantly associated with care recipient characteristics for either men or women. It was, by contrast, associated with several caregiver characteristics, type of care provision, and perceived effects of the pandemic.

After adjustment for age, women with a third-level education were less likely to describe being in poor health (OR: 0.15; CI: 0.03–0.81). Social support exerted a protective effect among male caregivers, as those with high support were 85% less likely to perceive poor health. This association was not significant among female caregivers, who were three times as likely to have poor self-perceived health if they had been diagnosed with COVID-19 in the past year. Overall, however, the number of women and men infected was very low.

Caregiving intensity had a strong influence on self-perceived health in the bivariate analyses. Both men and women who spent more than 14 h a day providing care were more likely to report poor health (OR: 4.16; CI: 1.00–17.24 and OR: 4.12; CI: 1.28–13.20, respectively). A high level of caregiver burden also increased the likelihood of poor self-perceived health, particularly in men, with an OR of 4.51 (CI: 1.49–13.63). The OR for women was 2.80 (CI: 1.30–6.03). Female caregivers who had support from families or close circles were half as likely to report poor health. This association was not significant in men.

Men felt the effects of the COVID-19 pandemic significantly more than women (Table 5). Increases in caregiving intensity and burden and worsening of finances and emotional wellbeing were significant predictors of poor self-perceived health among male caregivers, with ORs ranging between 3 and 5. The likelihood of a negative impact on health was also increased in women, but in this case, the associations were not significant.

The combined multivariate model showed a clear association between gender and self-perceived health (Table 6). After adjustment for all other variables, female caregivers were twice as likely as their male counterparts to report poor health (OR: 2.17; CI: 1.09–4.33). This combined analysis also highlights the strong association between certain caregiving characteristics and the perceived effects of the pandemic. Factors significantly associated with poor self-perceived health were a lower level of education, low social support, and increased caregiver burden.

The gender-stratified analysis also revealed some differences between male and female caregivers. A third-level education, for example, exerted a protective effect against poor self-perceived health in women (OR: 0.74; CI: 0.01–0.49), while in men, the protective effect was conferred by social support (OR: 0.06; CI: 0.01–0.34). An increased sensation of caregiver burden as a result of the pandemic was associated with a greater likelihood of poor self-perceived health in women (OR: 2.51; CI: 1.12–5.66) and especially men (OR: 5.84; CI: 1.33–25.66).

## 4. Discussion

We have investigated the effects of caregiving on informal caregivers during the COVID-19 pandemic from a gender perspective. Very few such studies have been conducted worldwide, and none in Spain. Our findings thus provide novel insights into caregiver perceptions of the effects of the pandemic on care provision and health and highlights possible implications for gender equality.

The results of our survey, performed 1 year into the pandemic, confirm that men and women have different perceptions of how the pandemic has directly affected their caregiving situation, health, and other aspects of their lives. Women continue to shoulder the brunt of care, and their situation appears to have worsened. Our findings show that more female than male caregivers experienced increased caregiving intensity and burden and worse self-perceived health. It should, however, be noted that most highly burdened male caregivers providing high-intensity care also reported poor health, situating them in a similar position to female caregivers in nonpandemic times. Our findings also corroborate the positive effects of support on caregiver wellbeing. A lower proportion of men than women reported reductions in informal support during the pandemic. Their social support network remained more stable and exerted a protective effect against poor self-perceived health. Women, by contrast, noticed a reduction in both formal and informal support, and in this case, those with a higher level of education were less likely to report poor health. Our survey has shed light on crucial aspects of caregiving and shown that they are sensitive to gender and the effects of the pandemic.

As a result of the pandemic, women experienced a greater increase in caregiving intensity than men and more often performed personal care tasks and household chores without help. Prepandemic studies conducted both in our setting and elsewhere have reported similar gender differences in the performance of these tasks [31,32,33]. The persistence of traditional gender roles largely explains why women take more responsibility for household tasks, tasks that are less gratifying, and tasks that take a greater toll on health, while men tend more to look after tasks outside the home and tasks that have a less deleterious effect on health [31]. This tendency was confirmed during lockdown [34], and similar differences were detected in our cohort.

Our findings show that the pandemic intensified existing inequalities in care provision and that men and women perceived its effects differently. Both groups reported feeling considerably more burdened than before and also reported that their social relationships and leisure pursuits had been affected. Similar to recent studies [21], our results highlight the importance of increasing the visibility of the crucial work performed by informal caregivers during the pandemic, work that is essential to the core principles of any welfare state and that, in many cases, was performed in difficult circumstances with very little help. The pandemic had a greater impact on female caregivers, as not only did the number of hours spent caregiving (often without help) increase, but they were also more likely to experience a decline in emotional wellbeing. Little has been published on the effects of the pandemic on caregiver mental health, but findings so far have highlighted the vulnerability of this population and shown that the pandemic has negatively affected emotional wellbeing, especially in the case of younger people and women [35].

A recent study of pandemic-induced changes experienced by caregivers for people with dementia reported similar findings to ours and provided several important insights [12]. The authors found that both male and female caregivers experienced an increase in caregiving intensity, but the impact on caregiver burden and health was different, highlighting the complex nature of these changes and their differential effects. The multivariate models in our study showed worse self-perceived health among female caregivers, even after adjustment for other aspects of care provision. Similar findings have been reported elsewhere [31,36]. We believe that these differences may be linked to gender differences in coping strategies, the persistence of traditional gender norms that assign women the role of caregiver, and the force with which women still identify with and accept this role [37].

Gender analysis and the concept of hegemonic masculinity provide a framework for helping to understand why male caregivers who experienced an increase in burden and a strain on their finances as a result of the pandemic perceived negative health impacts. Men who experienced increases in caregiving intensity and burden were more likely than women to report poor health. Similar findings for other types of informal caregivers have been attributed to gender differences in resilience [12,38]. It has been suggested that female caregivers are more resilient than their male counterparts [39] and this resilience might increase at times of extreme stress and uncertainty [12]. Studies of formal caregivers have found that women cope better under stress than men [40]. There is also evidence that increases in anxiety and other stressors during the pandemic may have magnified the effects of care provision on caregivers who were already significantly burdened [12].

We believe that gender differences in approaches to caregiving and coping strategies must be taken into account and efforts made to ensure that nobody, regardless of gender, is left to deal with extreme situations of burden without the necessary support.

The positive effect of social support on health and wellbeing has been widely documented [41,42], and it has been shown that strong social networks can mitigate the negative effects of caregiving [25]. Reductions in support structures during the pandemic have had severe consequences for caregiver health [43]. Our results also show differences between men and women in this respect. Social support during the pandemic had a stronger impact on the emotional wellbeing of men, possibly because they felt the effects of lockdown more acutely than women as they have traditionally enjoyed greater access to public and social spaces. Previous waves of the CUIDAR-SE survey have detected gender-based and geographic differences in social support networks. Women from the southern province of Granada, for example, received more support from their immediate circles than men, while the opposite was true for Gipuzkoa, in northern Spain [44]. We did not analyze geographic differences in support received during the pandemic, but it would be interesting to explore these differences and their potential effect on caregiver health.

One particular strength of this study is that the cohort comprises different types of informal caregivers looking after people with very different needs. Another strength is that we included pandemic-specific stressors and other key contextual factors, such as caregiver–recipient relationship and type of caregiving work. Very few studies have taken these factors into account when analyzing the perceived effects of the COVID-19 pandemic [44], and even fewer have done so from a gender perspective.

We took several steps to address the methodological limitations of our study. First, its cross-sectional design prevents us from drawing any causal inferences, but the formulation of the questions about the effects of the pandemic enabled us to show that it did have a significant effect on the health and wellbeing of male and female caregivers. Second, although our findings cannot be extrapolated to Spain as a whole, the CUIDAR-SE cohort comprises caregivers from two regions that differ both socioeconomically and in terms of access to social care and support services for dependent people [24,45]. This helps understand the universality of gender determinants in different contexts. A final limitation of our study is that all the members of our cohort are registered caregivers. Nonetheless, we believe that caregivers who do not register with the authorities probably dedicate much less time to this activity. The caregiver profile in our study is thus that of a long-term caregiver providing high-intensity care. Our findings could be extrapolated to caregivers with a similar profile, who we believe should be a priority target for support interventions.

Gender-based research on the effects of the COVID-19 pandemic on caregiving is still limited. Our results provide key insights that could be useful for informing gender-based interventions aimed at supporting already stretched and burdened caregivers. More research, however, is needed. Much remains to be done, particularly in the current situation, to implement actions that will break the cycle of inequality, promote a greater sharing of responsibilities across the board, and increase access to formal support systems.

## 5. Conclusions

Gender differences in informal caregiving already existed before the pandemic, with women faring worse than men in terms of health and quality of life. Caregivers of both genders have played an essential role in caring for the most vulnerable members of our society during the pandemic. Our findings indicate that women experienced a greater increase in caregiving intensity, a greater reduction in informal support, and a greater decline in emotional wellbeing. Both men and women reported feeling more burdened than before the pandemic and also mentioned a significant impact on their social lives and leisure activities. Women’s health was affected more than men’s, even after correction for other aspects of caregiving. These findings should be taken into account to design gender-responsive actions aimed at providing adequate support to caregivers and reversing the impact of the pandemic, such that the urgency of the current situation does not eclipse what is important. Our results may be useful in considering the differential impacts of the pandemic on the health of women and men informal carers, both in the design of health policies and in clinical and social care for carers.

## Figures and Tables

**Table 1:** Caregiver and recipient characteristics according to caregiver gender.

Variable	Male Caregivers N = 96	Female Caregivers N = 165	Total N = 261	χ2
Caregiver Characteristics				
	n (%)	n (%)	n (%)	*p*
Province				
Granada	40 (41.7)	88 (53.3)	128 (49.0)	
Gipuzkoa	56 (58.3)	77 (46.7)	133 (51.0)	0.069
Caregiver age				
<45	5 (5.2)	11 (6.7)	16 (6.1)	
45–64	41 (42.7)	109 (66.1)	150 (57.5)	
≥65	50 (52.1)	45 (27.3)	95 (36.4)	<0.001
Educational level				
No schooling	11 (11.5)	12 (7.3)	23 (8.8)	
Primary	19 (19.8)	29 (17.7)	48 (18.5)	
Secondary	51 (53.1)	97 (59.1)	148 (56.9)	0.635
Third-level	15 (15.6)	26 (15.9)	41 (15.8)	
Occupational status				
In paid employment	21 (21.9)	46 (27.9)	67 (25.7)	
Not in paid employment	75 (78.1)	119 (72.1)	194 (74.3)	0.284
Living with a partner				
Yes	75 (78.1)	120 (72.7)	195 (74.7)	
No	21 (21.9)	45 (27.3)	66 (25.3)	0.333
Social support				
Low	26 (27.1)	42 (25.5)	68 (26.1)	0.773
High	70 (72.9)	123 (74.5)	193 (73.9)	
Self-reported general health				
Good	67 (69.8)	90 (54.9)	157 (60.4)	
Poor	29 (30.2)	74 (45.1)	103 (39.6)	0.018
**Care recipient characteristics**				
	**n (%)**	**n (%)**	**n (%)**	***p***
Care recipient age				
≤20	4 (4.2)	26 (15.8)	30 (11.5)	
21–40	3 (3.1)	21 (12.7)	24 (9.2)	
41–70	36 (37.5)	35 (21.2)	71 (27.2)	<0.001
71–85	32 (33.3)	35 (21.2)	67 (25.7)	
>85	21 (21.9)	48 (29.1)	69 (26.4)	
Relationship with caregiver				
Spouse/partner	58 (60.4)	48 (29.1)	106 (40.6)	
Child	10 (10.4)	50 (30.3)	60 (23.0)	
Parent	24 (25.0)	59 (35.8)	83 (31.8)	<0.001
Another type of relative	4 (4.2)	8 (4.8)	12 (4.5)	
Level of dependence				
Moderate	19 (21.1)	31 (19.5)	50 (20.1)	
Severe	25 (27.8)	48 (30.2)	73 (29.3)	0.907
Major	46 (51.1)	80 (50.3)	126 (50.6)	
Cognitive deterioration				
No	59 (61.5)	109 (66.1)	168 (64.4)	
Yes	37 (38.5)	56 (33.9)	93 (35.6)	0.454
Diagnosed with COVID-19				
No	91 (94.8)	161 (97.6)	252 (96.6)	
Yes	5 (5.2)	4 (2.4)	9 (3.4)	0.235

**Table 2:** Type of care, caregiving intensity, caregiver burden, and support systems according to caregiver gender.

Variable	Male Caregivers N = 96	Female Caregivers N = 165	Total N = 261	χ2
	n (%)	n (%)	n (%)	*p*
Personal care tasks				
Yes, with no help	43 (44.8)	100 (60.6)	143 (54.8)	
Yes, with help	42 (43.8)	49 (29.7)	91 (34.9)	
No	11 (11.5)	16 (9.7)	27 (10.3)	0.041
Care-related physical mobility tasks				
Yes, with no help	54 (56.3)	70 (42.4)	124 (47.5)	
Yes, with help	28 (29.2)	42 (25.5)	70 (26.8)	
No	14 (14.6)	53 (32.1)	67 (25.7)	0.007
Care-related household chores				
Yes, with no help	47 (49.0)	113 (68.5)	160 (61.3)	
Yes, with help	44 (45.8)	50 (30.3)	94 (36.0)	0.003
No	5 (5.2)	2 (1.2)	7 (2.7)	
Caregiving intensity (hours/day)				
<8	25 (35.7)	24 (20.5)	49 (26.2)	
8–14	32 (45.7)	62 (53.0)	94 (50.3)	
>14	13 (18.6)	31 (26.5)	44 (23.5)	0.064
Level of caregiver burden				
None (≤46)	54 (56.3)	75 (45.5)	129 (49.4)	
Mild (47–55)	19 (19.8)	43 (26.1)	62 (23.8)	
High (≥56)	23 (24.0)	47 (28.5)	70 (26.8)	0.235
Informal help with caregiving				
Yes	63 (65.6)	115 (69.7)	178 (68.2)	
No	33 (34.4)	50 (30.3)	83 (31.8)	0.496
Paid help with caregiving				
Yes	16 (16.7)	22 (13.3)	38 (14.6)	
No	80 (83.3)	143 (86.7)	223 (85.4)	0.287
PECF allowance				
Yes	43 (44.8)	90 (54.9)	133 (51.2)	
No	53 (55.2)	74 (45.1)	127 (48.8)	0.116
Home help services				
Yes	23 (24.0)	31 (18.8)	54 (20.7)	
No	73 (76.0)	134 (81.2)	207 (79.3)	0.320
Home medical and nursing services				
Yes	37 (38.5)	72 (43.6)	109 (41.8)	
No	59 (61.5)	93 (56.4)	152 (58.2)	0.421

**Table 3:** Direct effects of the pandemic on caregiving and personal circumstances as perceived by male and female caregivers.

Variable	Male Caregivers N = 96	Female Caregivers N = 165	Total N = 261	χ2
	n (%)	n (%)	n (%)	*p*
Increase in caregiving intensity (hours/day)				
No	70 (72.9)	92 (55.8)	162 (62.0)	
Yes	26 (27.1)	73 (44.2)	99 (37.9)	0.009
Increase in caregiver burden				
No	59 (61.5)	90 (54.9)	149 (57.3)	
Yes	37 (38.5)	74 (45.1)	111 (42.7)	0.301
Reduction in informal support				
No	76 (79.2)	102 (63.4)	178 (69.3)	0.008
Yes	20 (20.8)	59 (36.6)	79 (30.7)	
Reduction in formal support				
No	73 (80.2)	131 (81.9)	204 (81.3)	
Yes	18 (19.8)	29 (18.1)	47 (18.7)	0.747
Negative impact on emotional wellbeing ^1^				
No	42 (44.2)	42 (25.5)	84 (32.3)	
Yes	53 (55.8)	123 (74.5)	176 (67.7)	0.002
Negative impact on financial situation ^1^				
No	83 (87.4)	142 (86.1)	225 (86.5)	
Yes	12 (12.6)	23 (13.9)	35 (13.5)	0.766
Negative impact on family relationships ^1^				
No	71 (74.0)	110 (67.1)	181 (69.6)	
Yes	25 (26.0)	54 (32.9)	79 (30.4)	0.244
Negative impact on social life and leisure activities ^1^				
No	48 (50.0)	78 (47.3)	126 (48.3)	
Yes	48 (50.0)	87 (52.7)	135 (51.7)	0.671
Diagnosed with COVID-19 (caregiver) ^2^				
No	88 (91.7)	154 (93.3)	242 (92.7)	
Yes	8 (8.3)	11 (6.7)	19 (7.3)	0.617

^1^ Refers to perception of a substantial change (including “changed a lot or quite a lot” and excluding “changed a little”); ^2^ Variable excluded from the multivariate analysis due to low prevalence (in <10 men).

**Table 4:** Bivariate analysis of poor self-perceived health according to caregiver, care recipient, and caregiving characteristics. Prevalences and odds ratios (ORs) adjusted for age and stratified by gender.

Poor Self-Perceived Health According to Caregiver Characteristics						
	N (%)	OR (95% CI)	*p*			
Gender						
Male		1				
Female		1.96 (1.28–3.39)	0.017			
		**Men**			**Women**	
	**N (%)**	**OR (95% CI)**	***p***	**N (%)**	**OR (95% CI)**	***p***
Province						
Granada	15 (37.5)	1		45 (51.1)	1	
Gipuzkoa	14 (25.0)	0.55 (0.22–1.37)	0.199	29 (38.2)	0.58 (0.31–1.09)	0.092
Educational level						
No schooling	4 (36.4)	1		6 (50.0)	1	
Primary	7 (36.8)	1.01 (0.21–4.78)	0.989	15 (51.7)	0.99 (0.25–3.92)	0.996
Secondary	16 (31.4)	0.78 (0.17–3.46)	0.738	48 (50.0)	0.88 (0.24–3.16)	0.841
Third-level	2 (13.3)	0.26 (0.04–1.95)	0.191	4 (15.4)	0.15 (0.03–0.81)	0.027
Occupational status						
In paid employment	6 (28.6)	1		19 (42.2)	1	
Not in paid employment	23 (30.7)	1.04 (0.30–3.63)	0.949	55 (46.2)	1.15 (0.54–2.45)	0.723
Living with a partner						
Yes	23 (30.7)	1		56 (46.7)	1	
No	6 (28.6)	0.95 (0.30–3.01)	0.924	18 (40.9)	0.79 (0.39–1.60)	0.516
Social support						
Low	15 (57.7)	1		23 (54.8)	1	
High	14 (20.0)	0.15 (0.05–0.44)	<0.001	51 (41.8)	0.57 (0.27–1.17)	0.122
Diagnosed with COVID-19 (caregiver)						
No	28 (31.8)	1		66 (43.1)	1	
Yes	1 (12.5)	0.31 (0.04–2.67)	0.286	8 (72.7)	3.89 (0.97–15.71)	0.056
**Poor self-perceived health according to care recipient characteristics**						
		**Men**			**Women**	
	**N (%)**	**OR (95% CI)**	***p***	**N (%)**	**OR (95% CI)**	***p***
Care recipient age						
≤40 y	2 (28.6)	1		21 (44.7)	1	
41–85 y	20 (29.4)	0.98 (0.17–5.74)	0.983	34 (48.6)	1.11 (0.49–2.53)	0.799
>85 y	7 (33.3)	1.21 (0.18–7.98)	1.209	19 (40.4)	0.81 (0.34–1.93)	0.626
Relationship with caregiver						
Spouse/partner	18 (31.0)	1		24 (50.0)	1	
Child	3 (30.0)	1.03 (0.21–5.02)	0.967	21 (42.0)	0.75 (0.32–1.79)	0.518
Parent	7 (29.2)	1.01 (0.28–3.63)	0.985	26 (44.8)	0.84 (0.37–1.88)	0.664
Level of dependence (perceived by caregiver)						
Moderate	4 (21.1)	1		16 (51.6)	1	
Severe	7 (28.0)	1.45 (0.36–5.95)	0.603	17 (35.4)	0.52 (0.21–1.29)	0.157
Major	15 (32.6)	1.82 (0.51–6.48)	0.354	36 (45.6)	0.78 (0.34–1.80)	0.562
Cognitive deterioration						
No	16 (27.1)	1		47 (43.1)	1	
Yes	13 (35.1)	1.46 (0.60–3.53)	0.406	27 (49.1)	1.26 (0.64–2.45)	0.504
Care recipient or another person in the house diagnosed with COVID-19						
No	26 (29.5)	1		64 (43.5)	1	
Yes	3 (37.5)	1.42 (0.32–6.39)	0.648	10 (58.8)	1.91 (0.68–5.33)	0.218
**Poor self-perceived health according to caregiving characteristics**						
		**Men**			**Women**	
	**N (%)**	**OR (95% CI)**	***p***	**N (%)**	**OR (95% CI)**	***p***
Personal care tasks						
Yes, without help	11 (25.6)	1		43 (43.4)	1	
Yes, with help	16 (38.1)	1.78 (0.70–4.50)	0.223	24 (49.0)	1.25 (0.63–2.49)	0.521
No	2 (18.2)	0.64 (0.12–3.45)	0.607	7 (43.8)	1.02 (0.35–2.97)	0.967
Care-related mobility tasks						
Yes, without help	15 (27.8)	1		26 (37.7)	1	
Yes, with help	10 (35.7)	1.44 (0.54–3.83)	0.462	22 (52.4)	1.82 (0.83–3.95)	0.133
No	4 (28.6)	1.04 (0.28–3.82)	0.958	26 (49.1)	1.63 (0.78–3.41)	0.191
Care-related household chores						
Yes, without help	13 (27.7)	1		49 (43.8)	1	0.818
Yes, with help	13 (29.5)	1.10 (0.44–2.74)	0.834	23 (46.0)	1.08 (0.55–2.12)	
No	3 (60.0)	4.35 (0.62–30.52)	0.139	2 (100.0)	---	
Hours spent caregiving a day						
<8	7 (28.0)	1		7 (29.2)	1	
8–14	6 (18.8)	0.57 (0.16–2.00)	0.378	26 (42.6)	1.79 (0.65–4.95)	0.262
>14	8 (61.5)	4.16 (1.00–17.24)	0.050	19 (61.3)	4.12 (1.28–13.20)	0.017
Level of caregiver burden						
None	11 (31.4)	1		37 (39.8)	1	
Mild	6 (21.4)	1.27 (0.38–4.28)	0.697	19 (55.9)	2.14 (0.98–4.65)	0.055
High	12 (36.4)	4.51 (1.49–13.67)	0.008	18 (48.6)	2.80 (1.30–6.03)	0.009
Informal support						
No	10 (30.3)	1		28 (57.1)	1	
Yes	19 (30.2)	1.02 (0.40–2.61)	0.968	46 (40.0)	0.50 (0.25–0.99)	0.048
Paid help with caregiving						
No	22 (27.2)	1		60 (42.6)	1	
Yes	7 (46.7)	2.37 (0.75–7.55)	0.144	14 (60.9)	2.08 (0.84–5.16)	0.113
PECF allowance						
No	20 (37.7)	1		37 (50.7)	1	
Yes	9 (20.9)	0.43 (0.17–1.10)	0.079	37 (41.1)	0.68 (0.36–1.26)	0.221
Home help services						
No	19 (26.0)	1		56 (41.8)	1	
Yes	10 (43.5)	2.35 (0.81–6.77)	0.114	18 (60.0)	2.08 (0.92–4.68)	0.078
Home medical and nursing services						
No	18 (30.5)	1		40 (43.5)	1	
Yes	11 (29.7)	0.96 (0.39–2.35)	0.920	34 (47.2)	1.15 (0.62–2.15)	0.660

**Table 5:** Bivariate analysis of self-perceived health according to direct effects of the pandemic on caregiving and personal circumstances as perceived by male and female caregivers. Prevalences and odds ratios (ORs) adjusted for age and stratified by gender.

		Male Caregivers			Female Caregivers	
	N (%)	OR (95% CI)	*p*	N (%)	OR (95% CI)	*p*
Increase in caregiving intensity (hours/day)						
No	16 (22.9)	1		41 (45.1)	1	
Yes	13 (50.0)	3.65 (1.37–9.71)	0.010	33 (45.2)	1.02 (0.55–1.92)	0.941
Increase in caregiver burden						
No	11 (18.6)	1		36 (40.4)	1	
Yes	18 (48.6)	4.45 (1.73–11.45)	0.002	38 (51.4)	1.65 (0.87–3.16)	0.128
Reduction in informal support						
No	24 (31.6)	1		41 (40.2)	1	
Yes	5 (25.0)	0.73 (0.24–2.26)	0.586	30 (51.7)	1.61 (0.83–3.09)	0.157
Reduction in formal support						
No	23 (31.5)	1		59 (45.4)	1	
Yes	5 (27.8)	0.85 (0.27–2.70)	0.788	12 (41.4)	0.85 (0.38–1.93)	0.702
Negative impact on emotional wellbeing ^1^						
No	7 (16.7)	1		15 (36.6)	1	
Yes	22 (41.5)	3.62 (1.35–9.69)	0.010	59 (48.0)	1.67 (0.80–3.53)	0.175
Negative impact on financial situation						
No	22 (26.5)	1		62 (44.0)	1	
Yes	7 (58.3)	4.20 (1.17–15.06)	0.028	12 (52.2)	1.47 (0.59–3.64)	0.408
Negative impact on family relationships						
No	18 (25.4)	1		51 (46.8)	1	
Yes	11 (44.0)	2.36 (0.90–6.16)	0.080	22 (40.7)	0.79 (0.40–1.56)	0.496
Negative impact on social life and leisure activities						
No	13 (27.1)	1		40 (51.9)	1	
Yes	16 (33.3)	1.40 (0.57–3.44)	0.461	34 (39.1)	0.59 (0.31–1.12)	0.106

**Table 6:** Multivariate models (combined and gender-stratified). Odds ratios (ORs) for poor self-perceived health according to explanatory variables.

	**Combined Gender Model**		**Male Caregiver Model**		**Female Caregiver Model**	
	**OR (95% CI)**	***p***	**OR (95% CI)**	***p***	**OR (95% CI)**	***p***
Gender						
Male	1		---	---	---	---
Female	2.17 (1.09–4.33)	0.028				
Age of caregiver (years, continuous variable)	1.00 (0.97–1.03)	0.899	0.99 (0.94–1.07)	0.980	0.99 (0.96–1.03)	0.736
Educational level						
No schooling	1		1		1	
Primary	1.20 (0.39–3.73)	0.756	2.17 (0.29–16.47)	0.453	1.14 (0.25–5.28)	0.871
Secondary	0.61 (0.21–1.78)	0.362	0.42 (0.05–3.39)	0.417	0.69 (0.16–3.02)	0.621
Third-level	0.09 (0.02–0.37)	0.001	0.18 (0.01–3.52)	0.256	0.74 (0.01–0.49)	0.007
Social support						
Low	1		1		1	
High	0.38 (0.19–0.79)	0.009	0.06 (0.01–0.34)	0.001	0.74 (0.31–1.77)	0.499
Relationship with caregiver						
Spouse/partner	1		1		1	
Child	1.03 (0.42–2.53)	0.947	1.11 (0.11–11.30)	0.929	0.93 (0.34–2.60)	0.896
Parent	0.74 (0.33–1.70)	0.481	0.20 (0.02–1.79)	0.150	0.79 (0.29–2.12)	0.641
Cognitive deterioration						
No	1		1		1	
Yes	1.03 (0.54–1.99)	0.923	1.33 (0.37–4.76)	0.666	0.97 (0.42–2.25)	0.939
Personal care tasks						
Yes, without help	1		1		1	
Yes, with help	1.93 (0.97–3.84)	0.061	3.72 (0.88–15.81)	0.075	1.66 (0.71–3.88)	0.243
No	1.49 (0.53–4.20)	0.454	1.43 (0.16–12.59)	0.750	1.60 (0.45–5.62)	0.466
Increase in caregiving intensity (hours/day)						
No	1		1		1	
Yes	1.01 (0.50–2.04)	0.972	2.51 (0.54–11.76)	0.243	0.71 (0.31–1.64)	0.425
Increase in caregiver burden						
No	1		1		1	
Yes	2.73 (1.42–5.26)	0.003	5.84 (1.33–25.66)	0.020	2.51 (1.12–5.66)	0.026
Negative impact on financial situation						
No	1		1		1	
Yes	2.18 (0.91–5.20)	0.081	3.05 (0.43–21.41)	0.262	1.72 (0.58–5.12)	0.327
Reduction in informal support						
No	1		1		1	
Yes	1.04 (0.51–2.11)	0.925	0.26 (0.04–1.73)	0.165	1.67 (0.71–3.92)	0.240
Reduction in formal support						
No	1		1		1	
Yes	0.85 (0.38–1.90)	0.696	0.65 (0.10–4.46)	0.663	0.84 (0.31–2.30)	0.737

## Data Availability

The data belong to the CUIDAR-SE survey and are owned by the Escuela Andaluza de Salud Pública and governed by this institute’s regulations as they contain potentially sensitive personal information. People interested in viewing these data can contact María del Mar García Calvente, main researcher of the CUIDAR-SE Study. Escuela Andaluza de Salud Pública. Cuesta del Observatorio, 4, 18011 Granada. Spain. E-mail: mariadelmar.garcia.easp@juntadeandalucia.es.

## References

[1] Wenham C., Smith J., Morgan R., Gender and COVID-19 Working Group (2020). COVID-19: The gendered impacts of the outbreak. Lancet.

[2] Castellanos-Torres E., Tomás-Mateos J., Chilet-Rosell E. (2020). COVID-19 en clave de género. Gac. Sanit..

[3] Bathia A. ONU Mujeres. Las Mujeres y el COVID-19: Cinco Acciones que los Gobiernos Pueden Adoptar sin Demoras. https://www.unwomen.org/es/news/stories/2020/3/news-women-and-covid-19-governments-actions-by-ded-bhatia.

[4] UN Women (2020). Policy Brief: The Impact of COVID-19 on Women.

[5] Eurofound (2020). Living, working and COVID-19. COVID-19 Series.

[6] European Institute for Gender Equality (EIGE) (2021). Gender Equality and the Socio-Economic Impact of the COVID-19 Pandemic.

[7] Connor J., Madhavan S., Mokashi M., Amanuel H., Johnson N.R., Pace L.E., Bartz D. (2020). Health risks and outcomes that disproportionately affect women during the Covid-19 pandemic: A review. Soc. Sci. Med..

[8] Vindegaard N., Benros M.E. (2020). COVID-19 pandemic and mental health consequences: Systematic review of the current evidence. Brain Behav. Immun..

[9] Collins C., Landivar L.C., Ruppanner L., Scarborough W.J. (2021). COVID-19 and the Gender Gap in Work Hours. Gend Work Organ.

[10] Johnston R.M., Mohammed A., van der Linden C. (2020). Evidence of Exacerbated Gender Inequality in Child Care Obligations in Canada and Australia during the COVID-19 Pandemic. Politics Gend.

[11] National Collaborating Centre for Methods and Tools (2020). Rapid Review: What Risk Factors Are Associated with COVID-19 Outbreaks and Mortality in Long-Term Care Facilities and What Strategies Mitigate Risk?.

[12] Cohen S.A., Kunicki Z.J., Drohan M.M., Greaney M.L. (2021). Exploring Changes in Caregiver Burden and Caregiving Intensity due to COVID-19. Gerontol. Geriatr. Med..

[13] European Commission (2021). Directorate-General for Employment, Social Affairs and Inclusion. Study on Exploring the Incidence and Costs of Informal Long-Term Care in the EU.

[14] Blue Cross Blue Shield (2020). The Impact of Caregiving on Mental and Physical Health. In The Health of America Report; Harnessing Data, for the Health of America. https://www.bcbs.com/sites/default/files/file-attachments/health-of-america-report/HOA-Caregivers_3.pdf.

[15] Park S.S. (2021). Caregivers’ Mental Health and Somatic Symptoms during COVID-19. J. Gerontol. B Psychol. Sci. Soc. Sci..

[16] Carpinelli-Mazzi M., Iavarone A., Musella C., De Luca M., de Vita D., Branciforte S., Coppola A., Scarpa R., Raimondo S., Sorrentino S. (2020). Time of isolation, education and gender influence the psychological outcome during COVID-19 lockdown in caregivers of patients with dementia. Eur. Geriatr. Med..

[17] Savla J., Roberto K.A., Blieszner R., McCann B.R., Hoyt E., Knight A.L. (2021). Dementia Caregiving During the “Stay-at-Home” Phase of COVID-19 Pandemic. J. Gerontol. B Psychol. Sci. Soc. Sci..

[18] Zucca M., Isella V., Lorenzo R.D., Marra C., Cagnin A., Cupidi C., Bonanni L., Laganà V., Rubino E., Vanacore N. (2021). Being the Family Caregiver of a Patient With Dementia During the Coronavirus Disease 2019 Lockdown. Front. Aging Neurosci..

[19] Suzuki K., Numao A., Komagamine T., Haruyama Y., Kawasaki A., Funakoshi K., Fujita H., Suzuki S., Okamura M., Shiina T. (2021). Impact of the COVID-19 Pandemic on the Quality of Life of Patients with Parkinson’s Disease and Their Caregivers: A Single-Center Survey in Tochigi Prefecture. J. Parkinsons Dis..

[20] Frangiosa T., Biggar V., Comer M., Roniger A. (2020). Research Survey Series Shows Effects of COVID-19 Shutdowns on Alzheimer’s Community, with Especially High Stress on Caregivers. Adv. Geriatr. Med. Res..

[21] Lorenz-Dant K., Comas-Herrera A. (2021). The Impacts of COVID-19 on Unpaid Carers of Adults with Long-Term Care Needs and Measures to Address these Impacts: A Rapid Review of Evidence up to November 2020. J. Long-Term Care.

[22] OECD (2018). Care Needed: Improving the Lives of People with Dementia. OECD Health Policy Studies.

[23] Mosquera-Metcalfe I., Larrañaga-Padilla I., Del Río-Lozano M., Calderón-Gómez C., Machón-Sobrado M., García-Calvente M.M. (2019). Desigualdades de género en los impactos del cuidado informal de mayores dependientes en Gipuzkoa: Estudio CUIDAR-SE. Rev. Esp. Salud Pública.

[24] Peña-Longobardo L.M., Del Río-Lozano M., Oliva-Moreno J., Larrañaga-Padilla I., García-Calvente M.M. (2021). Health, Work, and Social Problems in Spanish Informal Caregivers: Does Gender Matter? (The CUIDAR-SE Study). Int. J. Environ. Res. Public Health.

[25] Del Río-Lozano M., García-Calvente M.M., Calle-Romero J., Machón-Sobrado M., Larrañaga-Padilla I. (2017). Health-related quality of life in Spanish informal caregivers: Gender differences and support received. Qual. Life Res..

[26] García-Calvente M.M., Del Río-Lozano M., Ocaña-Riola R., Larrañaga-Padilla I., Mosquera-Metcalfe I. (2018). Evolución en el tiempo de la sobrecarga en mujeres y hombres cuidadores: Estudio CUIDAR-SE. Gac. Sanit..

[27] Rodríguez-Madrid M.N., Del Río-Lozano M., Fernández-Peña R., Jiménez-Pernett J., García-Mochón L., Lupiáñez-Castillo A., García-Calvente M.M. (2019). Gender Differences in Social Support Received by Informal Caregivers: A Personal Network Analysis Approach. Int. J. Environ. Res. Public Health.

[28] Moral-Fernández L., Frías-Osuna A., Moreno-Cámara S., Palomino-Moral P.A., Del-Pino-Casado R. (2018). Primeros momentos del cuidado: El proceso de convertirse en cuidador de un familiar mayor dependiente. Aten Primaria.

[29] Oliva-Moreno J., Peña-Longobardo L.M., García-Mochón L., Del Río-Lozano M., Mosquera-Metcalfe I., García-Calvente M.M. (2019). The economic value of time of informal care and its determinants (The CUIDARSE Study). PLoS ONE.

[30] (2006). Ley 39/2006, de 14 de Diciembre, de Promoción de la Autonomía Personal y Atención a las Personas en Situación de Dependencia (Promotion of Personal Autonomy and Care for Dependent People Act). BOE num.299. https://www.boe.es/eli/es/l/2006/12/14/39/con.

[31] García-Calvente M.M., Del Río-Lozano M., Marcos-Marcos J. (2011). Desigualdades de género en el deterioro de la salud como resultado del cuidado informal en España. Gac. Sanit..

[32] Cohen G., Russo M.J., Campos J.A., Allegri R.F. (2020). Living with dementia: Increased level of caregiver stress in times of COVID-19. Int. Psychogeriatr..

[33] Navaie-Waliser M., Spriggs A., Feldman P.H. (2002). Informal caregiving: Differential experiences by gender. Med. Care.

[34] Farre L., González L. Quien se Encarga de las Tareas Domésticas Durante el Confinamiento? Covid-19, Mercado de trabajo y uso del Tiempo en el Hogar. Fundacion BBVA. https://nadaesgratis.es/admin/quien-se-encarga-de-las-tareas-domesticas.

[35] Bennett M.R., Zhang Y., Yeandle S. (2020). Caring and COVID-19: Hunger and mental wellbeing. Sustainable Care: Care Matters 2020/01.

[36] Rosdinom R., Zarina M.Z., Zanariah M.S., Marhani M., Suzaily W. (2013). Behavioural and psychological symptoms of dementia, cognitive impairment and caregiver burden in patients with dementia. Prev. Med..

[37] Del Río-Lozano M., García-Calvente M.M., Marcos-Marcos J., Entrena-Durán F., Maroto-García G. (2013). Gender identity in informal care: Impact on health in Spanish Caregivers. Qual. Health Res..

[38] Schrank B., Ebert-Vogel A., Amering M., Masel E.K., Neubauer M., Watzke H., Zehetmayer S., Schur S. (2016). Gender differences in caregiver burden and its determinants in family members of terminally ill cancer patients. Psychooncology.

[39] Gaugler J.E., Kane R.L., Newcomer R. (2007). Resilience and transitions from dementia caregiving. J. Gerontol. B Psychol. Sci. Soc. Sci..

[40] Merlani P., Verdon M., Businger A., Domenighetti G., Pargger H., Ricou B., STRESI+ Group (2011). Burnout in ICU caregivers: A multicenter study of factors associated to centers. Am. J. Respir. Crit. Care Med..

[41] Matud M.P., García M.C., Fortes D. (2019). Relevance of Gender and Social Support in Self-Rated Health and Life Satisfaction in Elderly Spanish People. Int. J. Environ. Res. Public Health.

[42] Siedlecki K.L., Salthouse T.A., Oishi S., Jeswani S. (2014). The relationship between social support and subjective well-being across age. Soc. Indic Res..

[43] Giebel C., Cannon J., Hanna K., Butchard S., Eley R., Gaughan A., Komuravelli A., Shenton J., Callaghan S., Tetlow H. (2021). Impact of COVID-19 related social support service closures on people with dementia and unpaid carers: A qualitative study. Aging Ment. Health.

[44] Beach S.R., Schulz R., Donovan H., Rosland A.M. (2021). Family Caregiving During the COVID-19 Pandemic. Gerontologist.

[45] Rodríguez-Madrid M.N., Del Río-Lozano M., Fernández-Peña R., Elizalde-Sagardia B., García-Calvente M.M. (2021). Redes personales de apoyo y cuidado informal: ¿diferencias por sexo y territorio? (estudio CUIDAR-SE II). Gac. Sanit..

